# Degarelix vs. leuprorelin for the treatment of prostate cancer in China: A cost-utility analysis

**DOI:** 10.3389/fpubh.2022.942800

**Published:** 2022-07-18

**Authors:** Jianzhou Yan, Caiyun Li, Xuefang Zhang, Luyan Cheng, Ruilin Ding, Lingli Zhang

**Affiliations:** ^1^School of International Pharmaceutical Business, China Pharmaceutical University, Nanjing, China; ^2^The Research Center of National Drug Policy and Ecosystem, China Pharmaceutical University, Nanjing, China; ^3^School of Pharmacy, Nanjing Medical University, Nanjing, China

**Keywords:** degarelix, leuprorelin, prostate cancer, cost-utility analysis, Chinese healthcare system

## Abstract

**Objective:**

To explore the cost-effectiveness of degarelix acetate for injection (degarelix) compared to leuprorelin in prostate cancer (Pca) castration treatment from Chinese healthcare system perspective.

**Methods:**

A Markov model, adapted from the one established in Finland was conducted for the cost-effectiveness analysis of degarelix and leuprorelin for Pca treatment. The main data were derived from global phase III clinical trials of degarelix (CS21), published study and expert surveys. Outcomes, utility and costs of prostate cancer patients were calculated on a 30-year time horizon. The CS21 study based population of intention-to-treat (ITT) population and three scenarios were modeled. Taking three times of the Gross domestic product (GDP) per capita (242,928 yuan, 2021) as the acceptable threshold for cost-effectiveness. One-way and probabilistic sensitivity analyses were performed on key parameters, including transition probabilities, costs, utility, and discount rate to test the robustness of the model.

**Results:**

Base case analysis for ITT population revealed that total costs of degarelix and leuprorelin were 566,226 yuan and 489,693 yuan, while the total quality-adjusted life years (QALYs) were 5.19 and 4.51 during the 30-year time horizon, resulting an incremental cost effectiveness ratio (ICER) of 112,674 yuan/QALY which was 1.39 times the GDP per capita, lower than willingness-to-pay level of three times the GDP per capita. The results for scenario analyses revealed that compared to leuprorelin, degarelix for Pca treatment in China was cost-effective. One-way sensitivity analysis showed that the model was most sensitive to price of 80 mg degarelix, utility of 1st-line therapy, hazard ratio of PSA recurrence, price of 3.75 mg leuprorelin, response rate of docetaxel per cycle, and discount rate of cost. In probabilistic sensitivity analysis, compared to leuprorelin, the probability of degarelix to be cost-effective was 53 and 81% for willingness-to-pay threshold of one and three times the GDP per capita.

**Conclusion:**

Compared to leuprorelin, degarelix for prostate cancer treatment is cost-effective. Moreover, scenario, one-way, and probabilistic sensitivity analyses revealed that the model was robust.

## Introduction

Prostate cancer (Pca) is an epithelial malignant tumor of the prostate. According to the 2016 WHO “Pathology and Genetics Tumors of the Urinary System and Male Genital Organs,” the pathological types of prostate cancer include the most common adenocarcinoma (acinar adenocarcinoma), intraductal carcinoma, urothelial carcinoma, and squamous cell carcinoma among others (1).

Prostate cancer is one of the most common malignancies of the male genitourinary system. Its risk factors include race, age, and heredity (2). According to GLOBCAN released by WHO in 2018, globally, Pca was the second most common male malignant tumor, second only to lung cancer. Incidence rates of prostate cancer exhibit significant geographical and racial differences. The United States, Northern and Western Europe, Australia as well as New Zealand are high-incidence areas, with a maximum incidence rate of 86.4/100,000, while Asia and North Africa are relatively low-incidence areas with a minimum incidence rate of 5/100,000 (3). Prostate cancer is particularly common in developed countries, with about 249,000 new cases reported in the United States in 2021, accounting for 13.1% of all new cancer cases (4). A study published in 2016 assessed the incidence and mortality rates of prostate cancer in major countries and regions around the world. Based on age-standardized rate per 100,000 analysis, France (123.3/100,000), Sweden (107.6/100,000), Australia (108.2/100,000), the United States (106.8/100,000) and other developed countries had higher prostate cancer incidences than India (7.1/100,000), Thailand (8.7/100,000) and other Asian countries (5). Data from the National Cancer Center shows that since 2008, prostate cancer is the most prevalent tumor of the male urinary system in China. In 2016, its incidence rate was 11.12/100,000, ranking sixth with regard to male malignant tumors, while its mortality rate was 4.85/100,000, ranking seventh among all male malignant tumors (6).

Prostate cancer treatment is associated with a heavy financial burden globally. In Italy, the direct medical costs of Pca ranged from € 196 million to € 228 million per year, accounting for 0.2% of the national health service expenditure in 2016, while in Canada, the total cost of metastatic castration-resistant Pca was $193,604,000, and the total cost of medical castration and bone-targeted therapy to maintain castration testosterone levels was $416,284,000 (7). In China, prostate cancer-associated disease burden exhibited an increasing trend. From 1990 to 2013, the disability adjusted of life years (DALYs), the years of life lost (YLL), and the years lost due to disability (YLD) as a result of prostate cancer increased by 30.66, 25.51 and 51.5 thousand persons per year, with an annual growth rate of 1.05, 1.04 and 1.07% (8).

Androgen-deprivation therapy (ADT) as a primary systemic therapy in advanced prostate cancer patients, or as a neoadjuvant/adjuvant therapy in combination with radiotherapy for localized or locally advanced prostate cancer, includes castration and antiandrogen therapy. Castration therapy can be divided into surgical castration (bilateral orchiectomy) and medical castration, including luteinizing hormone-releasing hormone (LHRH, also known as gonadotropin-releasing hormone or GnRH) agonists or antagonists (6). Degarelix, which was approved in 2018 by National Medical Products Administration, was the first and only gonadotropin-releasing hormone (GnRH) antagonist marketed in China for prostate cancer treatment. International multicenter phase III clinical trials (CS21 and CS21A) (9, 10) and Chinese phase III clinical trials (PANDA) (11) showed that during prostate cancer treatment, compared to the widely used GnRH agonists, degarelix could rapidly reduce testosterone levels to the target level by day 3, significantly improving the survival outcomes of patients without PSA recurrence, and its safety was good.

Therefore, we choose leuprorelin, the most widely used GnRH agonist as reference to explore the cost-effectiveness of degarelix. The cost-effectiveness of degarelix has been proved in UK and US (12, 13) and there are no cost-utility analyses of degarelix in China. We aim to explore the cost-effectiveness of degarelix in China from the Chinese healthcare system perspective.

## Methods and materials

### Model structure and settings

#### Adaptation of the cost-utility model

This study was an adaptation of the Finnish model into Chinese environment national treatment practices and costs (14).

The Finnish model was a Markov process model to perform a cost–utility analysis of degarelix as castration treatment for patients with prostate cancer compared to standard treatment with LHRH agonists with anti-androgen flare protection. In this study, the basic structure of the model remained unchanged, and the corresponding disease conversion performed according to actual treatment situations and actual medical expenses data in China.

#### Model structure adaptation

**Figure 1:**
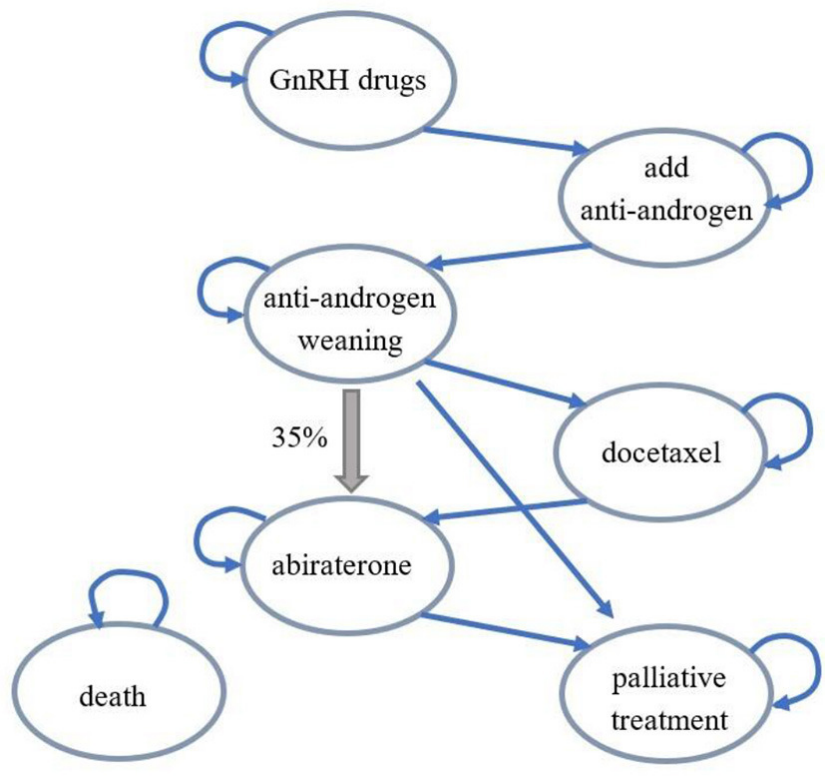
Structural diagram of the Chinese adaptation model.

With regard to prostate cancer treatment in China, the overall disease treatment process was different from the Finnish model. After anti-androgen withdrawal, 35% of patients would skip chemotherapy and accept abiraterone treatment (thick arrow). Therefore, during model adaptation, the transfer path from anti-androgen withdrawal to abiraterone was added to the disease conversion part. The modified structure diagram was as shown in Figure 1, and the disease state transitions were correspondingly added based on this structure diagram in the Chinese adaptation model. Each disease state in the model could progress to death state, and these transition arrows were not represented in the structure diagram to maintain aesthetics.

#### Comparator

Leuprorelin, the most widely used GnRH agonist in China, was selected as the reference. Leuprorelin acetate microsphere for injection (Etanercept, 3.75 mg) has been approved in China for nearly 30 years and is the main choice for androgen deprivation therapy. Degarelix is the only GnRH antagonist used for prostate cancer treatment, and the clinical Phase 3 trial (CS21) (9) evaluating the clinical efficacy and safety of degarelix had been completed with leuprorelin in combination with anti-androgen therapy (28 days) as the reference. 7.5 mg leuprorelin was selected as the reference in CS21 trial while this study chose 3.75 mg leuprorelin as the reference. The reasons for choosing 3.75 mg leuprorelin are as follows: (1) For leuprorelin, which has not yet been marketed in China with a specification of 7.5 mg, the most common specification in the market is 3.75mg; (2) The recommended usage and dosage in the drug instruction and CSCO Guideline for Diagnosis and Treatment of Prostate Cancer 2021 of the China Society of Clinical Oncology is 3.75 mg/4-week; (3) The HTA Review Report by All Wales Therapeutics and Toxicology Center (AWTTC) and study of degarelix conclude that there is no evidence of difference in the therapeutic efficacy of leuprorelin at doses of 7.5 and 3.75 mg (15, 16). Therefore, it is reasonable to use CS21 test data for the economic evaluation of leuprorelin with a lower dose of 3.75 mg.

### Simulated population

The population modeled was designed to reflect the participant population of the CS21 Phase III clinical trial (CS21) (9). The inclusion criteria of simulated population are as follows: (1) Men aged ≥18 years; (2) Histologically-confirmed adenocarcinoma of the prostate (any stage) for which endocrine treatment was indicated (except neoadjuvant hormonal therapy); (3) Increased PSA level despite previous treatment with curative intent; (4) Serum testosterone level of >1.5 ng/ml; (5) PSA level of ≥2 ng/ml; (6) ECOG score of ≤2. The exclusion criteria are as follows: (1) Candidate for curative therapy; (2) Previously or currently accepted hormonal management of prostate cancer (neoadjuvant or adjuvant hormonal therapy for localized treatment of curative intent was permitted if ≤6 months' duration and discontinued >6 months before study inclusion).

In addition to changes in model structure, domestic experts believe that survival time of domestic prostate cancer patients is not as long as that of European patients. The age of onset for domestic patients is lower than that of European patients (14, 17, 18). Therefore, the time horizon in the original Finnish model was set to 30 years, and the starting age of treatment changed from 72 to 68. The basic settings and some characteristics of the simulated population of the model were as shown in Table 1.

**Table 1:** The basic settings of the model.

**Parameters**	**Values**
Perspective	Chinese healthcare system
Targeted population	Intention to treat analysis (ITT), PSA >20 ng/ml
Comparator	Leuprorelin (3.75 mg) + anti-androgen bicalutamide (28 days)
Cycle	28 days
Time horizon	30 years
Discount rate	5%
Starting age	68 years old

### Treatment regimens

The first-line treatment regimens included in this study were degarelix acetate for injection and leuprorelin acetate microspheres for injection with anti-androgen flare protection. Specifications and drug regimens for each drug were:

Degarelix acetate for injection: 80 mg or 120 mg^*^2 sticks, according to the recommended dose of the drug insert (19), every 28 days as a medication cycle (Table 2).

**Table 2:** Medication regimen of degarelix acetate for injection.

**Starting dosage**	**Maintenance dosage**
240 mg given as two subcutaneous injections of 120 mg at a concentration of 40 mg/ml	80 mg given as one subcutaneous injection at a concentration of 20 mg/ml

The first maintenance dose should be given 28 days after the starting dose. Degarelix does not cause a surge in testosterone and does not require concomitant antiandrogen therapy for initial treatment.

Leuprorelin acetate microspheres: 3.75 mg, according to recommended dose in drug instructions and Guidelines of the Chinese Society of Clinical Oncology (CSCO) Prostatic Cancer 2021 (20), adults are subcutaneously administered with leuprorelin acetate (3.75 mg) every 4 weeks. Co-administration of anti-androgen drugs (28 days) with the first dose of leuprorelin should be done to avoid or reduce the testosterone “scintillation” effect.

### Model parameters

#### Efficacy

##### Clinical efficacy

Hazard ratios for PSA recurrence in the therapy with degarelix and leuprorelin were obtained from an open-label, multicenter, randomized, parallel-group study (CS21 trial). The global study involved prostate cancer patients and it evaluated the safety and efficacy of degarelix vs. leuprorelin (9).

The probability of a patient entering second-line therapy from first-line therapy was calculated based on the probability of progression on first-line therapy (PSA recurrence) and the probability of receiving various second-line therapy regimens after progression on first-line therapy. Regarding the progression probability of first-line therapy, since the period of the CS21 clinical trial was 12 months, clinical data for the 30-year study period of the model cannot be obtained. Therefore, we used the survival curve to simulate the survival data of patients outside the clinical trial period to obtain long-term efficacy data. Because the PSA recurrence in CS21 trial (9) was defined as two consecutive rises in PSA levels of 50% compared with nadir, and >5 ng/ml in two consecutive measurements at least 2 weeks apart. The Weibull, Loglogistic, Lognormal, Exponential, and Gompertz distributions were used to simulate and extrapolate the Kaplan–Meier curves of PSA progress of degarelix and leuprorelin. It was determined that PSA progress curve of leuprorelin group in Degenerate with the best fitting degree was the Loglogistic distribution curve according to the red pool information criterion (AIC). The survival function *S*(*t*) = 1/(1 + λ*t*^γ^) was Log-logistic distribution (*S* is the survival rate and *t* is the time), where γ and λ were shape and scale parameters of the Log-logistic function, *t* was time, and *S*(*t*) was the probability that a patient did not have PSA progression by time *t*.

**Figure 2:**
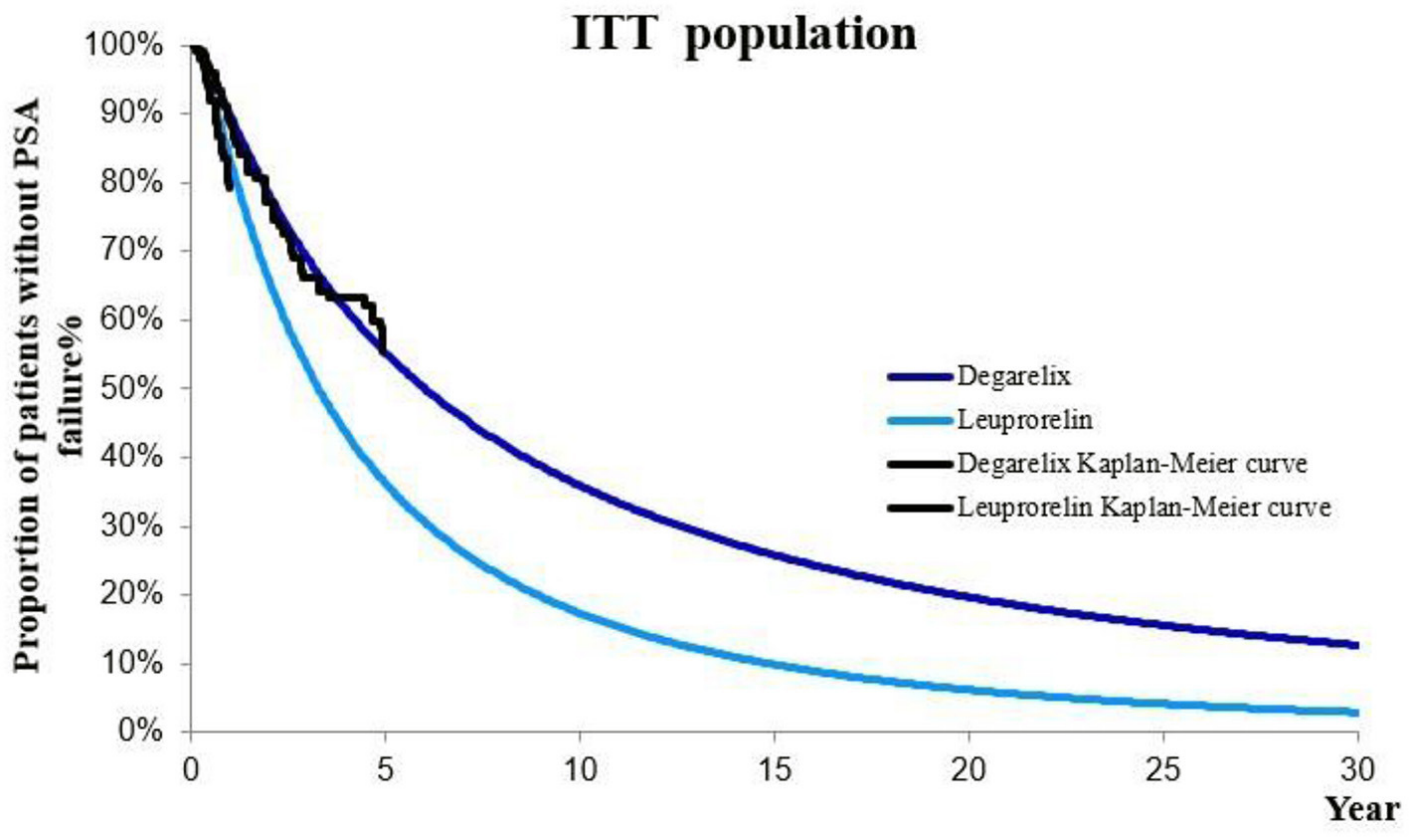
Kaplan–Meier curve of PSA progression in degarelix and leuprorelin groups (ITT population).

On this basis, we calculated the progression probability of first-line therapy for each cycle of patients in degarelix and leuprorelin groups. Corresponding probabilities for each cycle in the model were different (Figure 2).

Second-line therapy involved the anti-androgen addition, anti-androgen withdrawal, docetaxel, and abiraterone. Response rates for each therapy were presented in Table 3 (14).

**Table 3:** The response rates of second-line treatment.

**Type of treatment**	**Mean duration of response (months)**	**Proportion of patients %**	
		**Response rate per cycle**	**Non-response rate per cycle**
Add anti-androgens	6	0.83	0.17
Antiandrogen withdrawal	6	0.83	0.17
Docetaxel	12	0.91	0.09
Abiraterone	6.45	0.84	0.16

##### Mortality

The probability of transition from health state to death in the model was calculated from age-specific basal mortality and relative hazard ratio of the Chinese population by Logistic curve. Age-specific mortality rates of the Chinese population were obtained from the sixth census data (21). Mortality hazard ratio of PSA progression/metastatic was obtained from time-varying univariate and multivariate survival analyses of the relationship between PSA progression and survival in a previous study and the hazard ratio was 2.39 (22).

#### Cost

From the Chinese healthcare system perspective, we only considered direct medical costs, which were divided into the costs of: the drug, second-line treatment, treatment management, and adverse event treatment. Drug prices were the median prices of the latest ongoing bids, while treatment management and adverse event costs were mainly obtained from expert surveys. Due to large differences in medical care patterns in different regions and between urban and rural areas in China, as well as the fact that prostate cancer treatment is mainly concentrated in hospitals in large cities, the cost data for this study were mainly obtained from expert surveys of tertiary hospitals in first and second-tier cities. In this study, 16 experts at urology departments of tertiary hospitals in Beijing, Shanghai, Guangzhou, Chengdu and Xi'an were invited for questionnaire survey. Before conducting the survey, a deputy director expert in Beijing was invited to conduct a preliminary survey on question setting of the questionnaire. Then, after modifications had been made according to the opinions of the expert, interviews with other experts were conducted. The obtained data were sorted, analyzed and the mean calculated, which would be substituted into the model for corresponding calculations.

##### Prices of degarelix and leuprorelin

Degarelix prices in this study included the price of the first cycle high-dose and the price of the subsequent injection of each cycle, which were obtained from the winning bid database. The domestically marketed products of leuprorelin include the original drugs (Enantone, Takeda, 1,599.4 yuan/piece) and two generic drugs (Livzon 1,295.9 yuan/piece and Boente 1,272.58 yuan/piece). Therefore, the price was the average price of the original drugs and generic drugs (1,389.29 yuan/piece), which were obtained from the bid-winning database. Drug prices and dosages were as presented in Table 4.

**Table 4:** The price and dosage of each drug.

**Drug**	**Price (yuan)**	**Dosage**
Degarelix (120 mg^*^2 in the first cycle)	8,900	1
Degarelix (80 mg^*^1 in subsequent cycle)	3,200	Per cycle
Leuprorelin	1,389.29	Per cycle

##### Costs of disease management and other drugs

During prostate cancer treatment, in addition to the cost of degarelix and the corresponding reference substance, there are costs of disease management and other therapies. Drug costs were obtained from the latest implementation median price of the bid-winning data website in 2021, while the costs of medical resource consumption and disease management were obtained from expert survey (Table 5).

**Table 5:** Other treatment cost and administration cost.

**Parameters type**	**Parameters**	**Value**	**References**
Other treatment costs	Anti-androgen - flare cover/daily	¥169.37	Bid-winning data website
	Second-line enzalutamide 160mg/day (40 mg^*^4 capsules)	¥216.67	Bid-winning data website
	Docetaxel (20 mg)/daily	¥245.66	Bid-winning data website
	Abiraterone (250 mg)/daily	¥40.162	Bid-winning data website
	Dexamethasone (5 mg)/daily	¥0.07	Bid-winning data website
	Continued treatment after the failure of abiraterone/cycle	¥25,200.00	Expert survey
	Supportive care/cycle	¥7,500.92	Expert survey
	Palliative care/cycle	¥5,804.82	Expert survey
Administration Cost	GP consultation fee/each time	¥28.25	Expert survey
	Bone scan/each time	¥563.75	Expert survey
	CT scan/each time	¥457.50	Expert survey
	MRI/each time	¥1,440.00	Expert survey
	Blood test/each time	¥369.38	Expert survey

* *Drug price*.

*^≠^Drug cost*.

*^¥^Chinese currency*.

##### Costs of adverse event treatment

Adverse events in this study included cardiovascular events, musculoskeletal events, and spinal cord compression (SCC). Cardiovascular events were divided into fatal and non-fatal types; musculoskeletal events were divided into three types: mild, moderate, and severe, while spinal cord compression treatments were surgery and chemotherapy. All treatment costs were obtained from expert surveys (Table 6).

**Table 6:** Cost of adverse event management.

**Types of adverse event**		**Category**	**Value**
Spinal cord compression		Radiotherapy cost (per patient)	¥30,000.00
		Surgery fee (per patient)	¥40,000.00
Musculoskeletal events	Severe	Joint disorders	¥27,142.86
		Fracture	¥21,500.00
		Other musculoskeletal events	¥24,000.00
	Moderate	Joint disorders	¥16,276.50
		Fracture	¥15,276.72
		Other musculoskeletal events	¥17,776.50
	Mild	Joint disorders	¥14,528.25
		Fracture	¥7,417.14
		Other musculoskeletal events	¥8,278.25
Cardiovascular events	Fatal		¥28,723.96
	Non-fatal		¥18,369.28

^¥^*Chinese currency*.

#### Utility

Utility data in this study were obtained from the original Finnish model, which were from the study of Bayoumi et al. (23). Utility values of each disease state used in the model were as shown in Table 7.

**Table 7:** Utility values for each disease state.

**Entry**	**Value**
Utility of first-line treatment	0.90
Utility of anti-androgen addition	0.80
Utility of anti-androgen withdrawal	0.80
Utility of chemotherapy and abiraterone	0.69
Utility of supportive and palliative care	0.40

Based on the utility value for each health state, during drug treatment, we considered adverse event-associated changes in utility values for patients. We assumed that changes in utility values caused by each adverse event in degarelix and leuprorelin groups were equal, as shown in Table 8 (24, 25).

**Table 8:** Utility of adverse events.

**Adverse events**	**Value**
Severe SCC (non ambulant)	−0.20
Mild SCC (ambulant)	−0.37
Severe musculoskeletal events	−0.37
Moderate musculoskeletal events - applied as decrement	−0.26
Mild musculoskeletal events - applied as decrement	−0.12
Cardiovascular events	−0.73

### Base case analysis

Base case analysis results were expressed as incremental cost-effectiveness ratio (ICER) in ITT population, which was calculated as incremental costs/incremental QALYs. Based on the China Guidelines for Pharmacoeconomic Evaluations (26) recommendations, we used three times the GDP per capita as the threshold of Willingness to pay (WTP). If the ICER < the threshold of WTP, then, we can consider that the treatment strategy is cost effective. The GDP per capita was 80,976 yuan in China in 2021 (27).

### Scenario analyses

Scenario analyses were also conducted for different price of drug, time horizon, and dosage of drug to evaluate the economics of degarelix and leuprorelin for prostate cancer treatment.

### One-way sensitivity analysis

One-way sensitivity analysis was performed to examine the impact of variation in individual parameters on robustness of base-case results. The analysis was performed on model parameters such as hazard ratio of first-line treatment, response rate of second-line treatment, utility value of health states, cost and dosage of treatment drug, cost of second-line treatment drug, cost of treatment management, cost of best supportive care, and discount rate. Apart from reported varies in original parameters, without the source of parameters ranges, costs parameters varied between −20 and +20%, while efficacy parameters varied between −10 and +10%. Findings from one-way sensitivity analysis were measured by ICER, and the calculation formula was:


$$\begin{array}{l} \text{ICER }=\;\;({\text{Cost}}_{\text{degarelix arm}}\;-{\text{ Cost}}_{\text{leuprorelin arm}})/\\ \;({\text{QALYs}}_{\text{degarelix arm}}\;-{\text{QALYs}}_{\text{leuprorelin arm}}). \end{array}$$

If ICER are all less than zero, then, the intervention program is an absolute superiority program.

### Probabilistic sensitivity analysis

To verify the influence of parameter uncertainty as a whole on model results, probability sensitivity analysis was performed on the range of model parameters and their distribution characteristics, including hazard ratio of first-line treatment, response rate of second-line treatment, utility value of health state, cost and dosage of treatment drug, cost of second-line treatment drug, cost of treatment management, cost of best supportive care, and discount rate. Probabilistic sensitivity analysis was conducted by simultaneously varying all parameters within set different distributions in 5,000 Monte Carlo simulation iterations to illustrate the results of uncertain analysis and build a cost-effectiveness acceptability curve.

## Results

### Base case analysis

Using a 30-year time horizon Markov model (Table 9), the total costs for degarelix and leuprorelin groups were 566,266 yuan and 489,693 yuan, while total QALYs were 5.19 and 4.51, respectively. Compared to leuprorelin, incremental cost-effectiveness ratio of degarelix in prostate cancer treatment was 112,674 yuan/QALY, which was 1.39 times GDP per capita, lower than three times China GDP per capita in 2021. Therefore, degarelix was more economical for prostate cancer treatment than leuprorelin.

**Table 9:** Results of base case analysis.

**Treatment**	**Total cost (yuan)**	**QALYs**	**Incremental QALYs**	**ICER (yuan/QALY)**	**Incremental Net Benefit (INB)**
Degarelix	566,226	5.190	0.679	112,674	88,473
Leuprorelin	489,693	4.510			

### Scenario analyses

Scenario analyses simulated the cost-effectiveness of degarelix in the treatment of prostate cancer under three scenarios. Results of crowd scenario analyses were showed in Table 10. (1) Scenario 1: under the threshold of 1 time the GDP per capita, when the price of degarelix (80 mg) dropped to 2,968.59 yuan, a decrease of 7.23%, degarelix was economical for the treatment of prostate cancer. (2) Scenario 2: when the time horizon was 10 years, the ICER of degarelix for prostate cancer was 135,317 yuan/QALY, 1.67 times the GDP per capita. (3) Scenario 3: when the dose of leuprorelin was adjusted to 7.5 mg per cycle, the same with CS21 trial, the degarelix had a dominant advantage over the leuprorelin in the treatment of prostate cancer. Therefore, compared to leuprorelin, degarelix was more economical for prostate cancer treatment in China.

**Table 10:** Results of crowd scenario analyses.

**Scenario**	**Treatment solutions**	**Total cost (yuan)**	**QALYs**	**Incremental QALYs**	**ICER (yuan/QALY)**	**Incremental Net benefit value (INB)**
The price of degarelix was decreased by 7.23%	Degarelix	544,696	5.19	0.679	80,976	110,004
	Leuprorelin	489,693	4.51			
The time horizon was adjusted to 10 years	Degarelix	406,735	4.395	0.497	135,317	53,515
	Leuprorelin	339,442	3.898			
The dose of leuprorelin per cycle was adjusted to 7.5mg	Degarelix	566,266	5.19	0.679	Dominant	209,566
	Leuprorelin	610,786	4.51			

### One-way sensitivity analysis

Three times the GDP per capita was used as the WTP threshold to calculate ICER, then, a storm map was drawn based on one-way sensitivity analysis of degarelix relative to leuprorelin.

**Figure 3:**
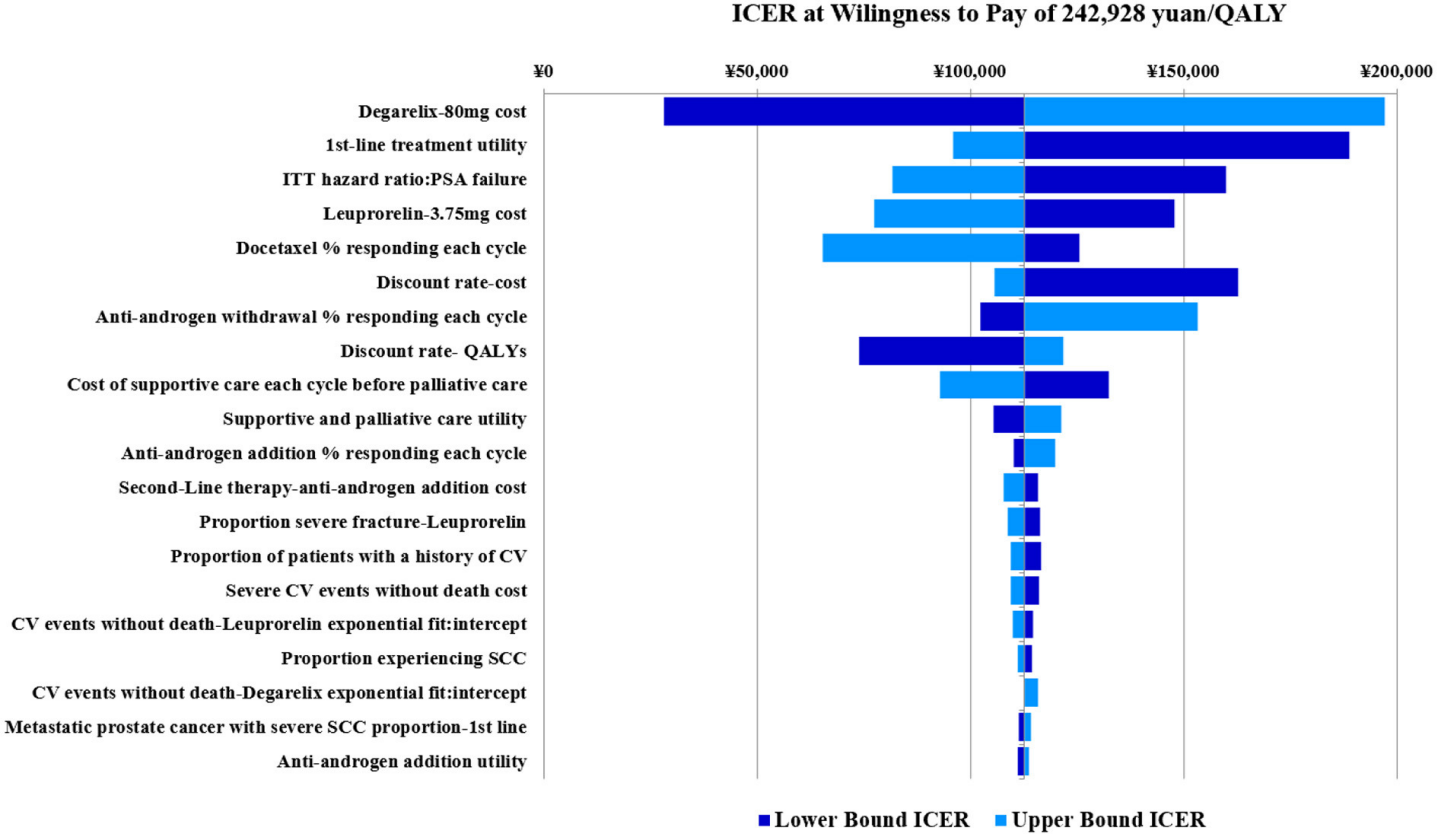
Degarelix vs. leuprorelin univariate sensitivity analysis tornado plot (base case analysis ICER= 112,674).

Figure 3 shows that when all uncertain factors changed within the specified range. Results of the one-way sensitivity analyses were largely consistent with those of the base-case analysis, with most showing limited variations from main results. Of all uncertainties, the six factors that had the greatest impact on outcomes were price of 80 mg degarelix, utility of 1st-line therapy, hazard ratio of PSA recurrence, price of 3.75 mg leuprorelin, response rate of docetaxel per cycle, and discount rate of cost. The other factors had limited effects. When the drug cost of degarelix 80 mg varied from 2,560 to 3,840 yuan, ICER fluctuated in the range of 28,252 to 197,096.

### Probabilistic sensitivity analysis

**Figure 4:**
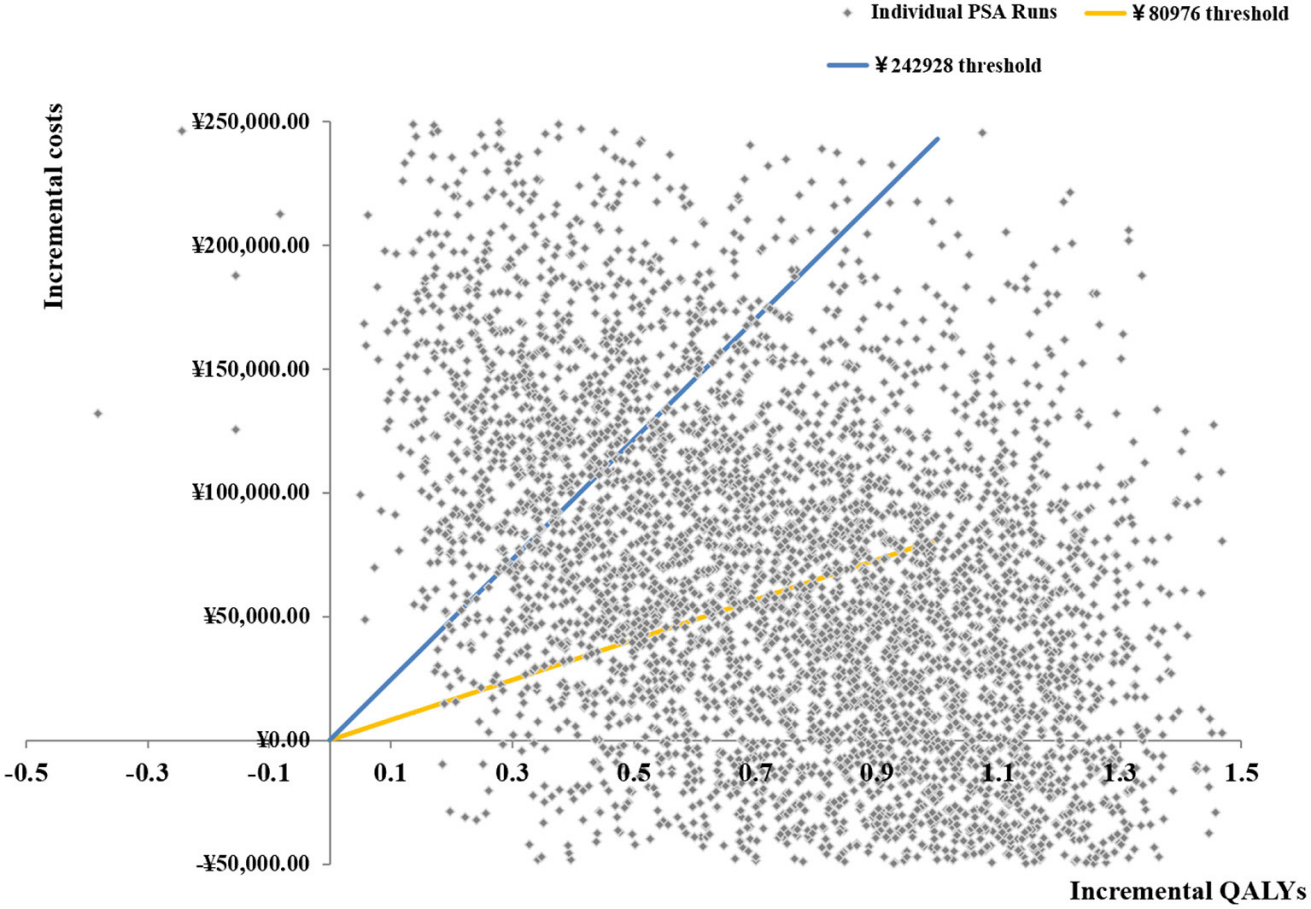
Base-case probabilistic sensitivity analysis: scatter plot (5,000 iterations).

**Figure 5:**
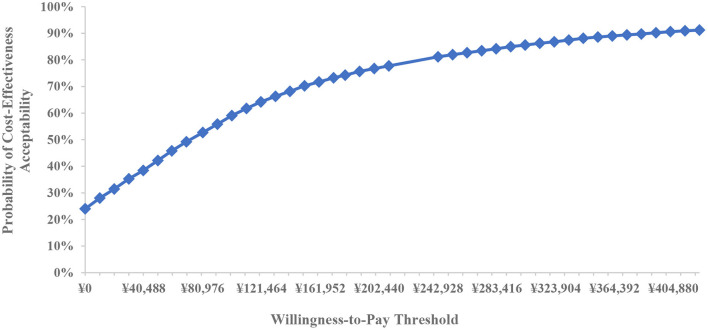
Cost-effectiveness acceptable curve.

Based on 5,000 Monte Carlo simulations, the cost-effectiveness scatterplot and the cost-effectiveness acceptable curve were drawn. From the cost-effect scatter plot (Figure 4), most of the scatter points were in the first quadrant of the coordinate axis, suggesting that degarelix could bring more QALYs, but at the same time, the cost was higher. When WTP was 112,674yuan/QALY, which is the ICER of base case analysis, the probability of degarelix having a cost-utility advantage was 62%. When WTP was one–three times the GDP per capita, most of the scattered points were below the threshold line. The cost-effectiveness acceptable curve (Figure 5) shows that when WTP was one–three times the GDP per capita, in the range of 80,976 to 242,928 yuan/QALY, the probability of degarelix having a cost-utility advantage was 53% and 81%.

## Discussion

This is the study that revealing the cost-effectiveness of degarelix in Chinese health system settings. The findings of our CEA modeling comparing degarelix with leuprorelin indicated that at the 30-year time horizon, degarelix was cost effective over leuprorelin as the baseline strategy at the threshold of three times the GDP per capita. One-way sensitivity analysis revealed that six factors that had the greatest impact on outcomes of degarelix compared to leuprorelin were price of 80 mg degarelix, utility of 1st-line therapy, hazard ratio of PSA recurrence, price of 3.75 mg leuprorelin, response rate of docetaxel per cycle, and discount rate of cost. Probabilistic sensitivity analysis showed that compared to leuprorelin, when WTP thresholds were one and three times the GDP per capita in 2021, probabilities of degarelix being cost-effective were 53 and 81%. The sensitivity analysis results revealed that our model was relatively robust. An additional threshold analysis indicated that the list price of degarelix would have to decrease by 7.23%, the cost of degarelix starter injections would be 8,098.11 yuan, and the cost of maintenance injections would be 2,968.59 yuan. The ICER will reach the threshold of 1 time Chinese GDP per capita (80,976 yuan, 2021). Our findings are in line with the previous results done in Pca patients from other national studies (12, 13).

Prostate cancer has a longer natural survival period than most other malignancies. At present, the main treatment methods for prostate cancer in the world mainly include radical surgery, radiation therapy, chemotherapy and endocrine therapy, and the survival time is the main indicator to evaluate the quality of various types of treatment. Endocrine therapy for prostate cancer has a history of more than 70 years. Endocrine therapy has been shown to effectively prolong the survival time of patients. In one study, the median survival time for prostate cancer patients treated with endocrine therapy was 7.81 years (28).

To date, only one research has evaluated the cost-effectiveness of degarelix and leuprorelin in the treatment of patients with Pca in China. Xuan et al. (14) evaluated the cost-effectiveness of degarelix, leuprorelin and goserelin from the perspective of Chinese healthcare system in 2018 and the conclusions were consistent with those of this study.

Degarelix is the third generation GnRH antagonist which can competitively block the GnRH receptor, resulting in a rapid, but reversible, decrease in LH, FSH and testosterone without any flare (29, 30). A meta-analysis study revealed that incidence rates of adverse reactions of degarelix were significantly lower than GnRH agonists, such as back pain, weight gain and cardiovascular events (31). And two initial studies that demonstrated this association analyzed data from the Surveillance Epidemiology and End Results-Medicare linked database and identified an increased risk of incident coronary heart disease, myocardial infarction, and cardiovascular death among men with prostate cancer treated with a GnRH agonist (32, 33). Therefore, degarelix can bring more clinical benefits to patients with Pca and the cost-effectiveness analysis is important for patients, clinicians and payers.

The major change in the process of adapting the Finnish model to China was the treatment cost. We obtained information and prices on clinical visits, medication, and health resource utilization in different disease states of prostate cancer by surveying urologists across the country. Drug prices were mainly obtained *via* the bid-winning data network. For instance, the price of abiraterone was the median of bid-winning prices of provinces and cities in the bid-winning data network. The number of visits, medical resource utilization, and cost per time of prostate cancer patients at each disease state were obtained from experts. Thus, the cost data in this study are subject to personal limitations.

The utility value in this study is obtained from the original Finnish model, which is the data of the European population. Due to limitations of domestic utility research, availability of utility data for each disease state of prostate cancer population is poor. Therefore, the original data was used in this study.

This study has several limitations. First the treatment management and adverse reaction costs in the model came from surveys of experts, which had a certain subjectivity. But treatment management and adverse reaction costs have great difference between hospitals, and no adequate cost data could be obtained from literature searches. On the other hand, prostate cancer treatment is mainly concentrated in hospitals in large cities, the cost data for this study were mainly obtained from expert surveys of tertiary hospitals in first and second-tier cities, which can match the real world level. Therefore, expert surveys were the best approaches for obtaining data. Second, regarding the probability of metastasis, due to a lack of data on response rates of prostate cancer patients in second-line treatment and best supportive care in China, data were obtained from international clinical trials. There may be cases where treatment effects of patients in mainland China differ from the results in clinical trials. However, there was no substitute for data that fully meets the modeling needs. If clinical data from patients in mainland China are available in future, we will replace the data in the existing model. Third there were no published studies on health preferences of various treatment regimens for Chinese prostate cancer patients, therefore, health utility values in the model were all taken from existing literature on international clinical trials. Since health utility values were greatly affected by country and race, if health utility values for Chinese prostate cancer patients under different treatment regimens can be directly obtained in future, we will replace the health utility value parameters in the existing model.

## Conclusion

This study used the Markov model to simulate the cost-effectiveness of degarelix vs. leuprorelin for prostate cancer treatment. During the 30-year simulation period, the study showed that compared to leuprorelin, degarelix had a cost-effective advantage in castration treatment of Chinese prostate cancer patients.

## Data availability statement

The raw data supporting the conclusions of this article will be made available by the authors, without undue reservation.

## Author contributions

JY and LZ designed the whole study. CL, XZ, LC, and RD collected and analyzed data. LC and RD conducted the models of the study. CL and XZ contributed to the original draft of the study and took the responsibility for review and editing. All authors contributed to the article and approved the submitted version.

## Funding

This work was supported by the National Social Science Foundation of China (15ZDB167).

## Conflict of interest

The authors declare that the research was conducted in the absence of any commercial or financial relationships that could be construed as a potential conflict of interest.

## Publisher's note

All claims expressed in this article are solely those of the authors and do not necessarily represent those of their affiliated organizations, or those of the publisher, the editors and the reviewers. Any product that may be evaluated in this article, or claim that may be made by its manufacturer, is not guaranteed or endorsed by the publisher.
